# Yeast Culture Improves Egg Quality and Reproductive Performance of Aged Breeder Layers by Regulating Gut Microbes

**DOI:** 10.3389/fmicb.2021.633276

**Published:** 2021-03-19

**Authors:** Yuchen Liu, Xue Cheng, Wenrui Zhen, Dan Zeng, Lujiang Qu, Zhong Wang, Zhonghua Ning

**Affiliations:** ^1^National Engineering Laboratory for Animal Breeding and Key Laboratory of Animal Genetics, Breeding and Reproduction, Ministry of Agriculture and Rural Affairs, College of Animal Science and Technology, China Agricultural University, Beijing, China; ^2^State Key Laboratory of Animal Nutrition, College of Animal Science and Technology, China Agricultural University, Beijing, China; ^3^Huayu Agricultural Science and Technology Co., Ltd., Handan, China

**Keywords:** yeast culture, aged layer, performance, egg quality, microbiome, reproduction

## Abstract

This study aimed to investigate the effects of dietary yeast culture (YC) supplementation on egg production, egg quality, reproductive performance, immune functions, antioxidant capacity, and intestinal microbial structure of aged hens. A total of 224 Hy-Line Brown layers (54 weeks old) were randomly assigned to two dietary treatments. The control group was fed a basal diet and the YC group was supplemented with YC at 2.0 g/kg of their diet. Each group had seven replicates with 16 hens each. The study was conducted over a period of 8 weeks. Results indicated that YC addition had no significant effect on laying performance. However, it significantly improved egg quality and hatching rate, enhanced ileum crude fat digestibility, increased the serum parameters of lysozyme (LZM) and total antioxidation capacity (T-AOC) (*P* < 0.05), and reduced serum aspartate aminotransferase (AST) levels (*P* < 0.05). Using 16S rRNA analysis, we found that addition of YC significantly altered ileum microbial composition. Linear discriminant analysis of effect size (LEfSe) showed significant enrichment of *Bacilli* and *Lactobacilli* in the YC group. PICRUSt analysis of the ileal microbiota found that glutathione metabolism, ubiquinone, and other terpenoid-quinone biosynthesis and lipopolysaccharide biosynthesis protein pathways were highly enriched in the YC group compared with the basal diet group. In summary, the addition of YC can improve egg quality, immune functions, antioxidant capacity, reproduction efficiency, and digestive absorption by increasing the abundance of *Lactobacilli* and *Bacilli*. Furthermore, it also improves the biosynthesis of lipopolysaccharide proteins, glutathione metabolism, and the synthesis of ubiquinone and other terpenoid-quinone metabolic pathways.

## Introduction

With an increase in large-scale layers, especially under conditions of high-density rearing, aged layers are susceptible to various stress factors that can lead to several problems such as imbalance in intestinal microbiota, reduction in antioxidant capacity (Liu et al., 2018), increase in immune inflammatory responses (Attia et al., 2020), and a decline in performance (Bar et al., 1999), egg quality (Bar et al., 1999), and reproductive efficiency (Liu et al., 2018). These changes lead to economic losses to the poultry industry. Although antibiotics are often used to control and treat pathogenic bacterial infections, such as those caused by pathogenic *Escherichia coli, Clostridium perfringens*, and *Salmonella* in aged hens, their use has been gradually banned during the laying period in many countries due to egg safety, antibiotic resistance, and environmental pollution issues (Wang et al., 2015). Thus, it is important and interesting to maintain gut health, enhance antioxidant activity, and delay senescence of aged hens through nutritional strategies, thus improving their laying performance and egg quality, and extending their lifespan.

Yeast culture (YC) is a type of microecological product produced under specific conditions of yeast fermentation. It contains yeast and various metabolites. YC is rich in proteins, small peptides, oligosaccharides, vitamins, minerals, enzymes and a variety of unknown growth factors which can provide abundant nutrition for gut microbes, stimulate the proliferation of beneficial bacteria and inhibit the growth of harmful bacteria (Liu et al., 2019). Several experiments have suggested that YC has a positive effect on layers and broilers. For example, Zhang J.C. et al. (2020) reported that YC addition at 3.0 g/kg of feed improves the performance of aged layers by upregulating intestinal digestive enzyme activity and intestinal health-related gene expression. Another study in broilers showed that dietary YC supplementation at 0.1 g/kg increases daily weight gain and reduces *Campylobacter* in the cecum (Froebel et al., 2019). In addition, yeast bioactive substances can alleviate the negative effects of *Eimeria* infection on the growth of poultry, improving the structure of the jejunum mucosa and increasing the content of IgA in the egg yolk (Lu et al., 2019). The addition of YC at 2.5 g/kg activates macrophages, thereby increasing lysozyme content in the serum of laying hens, thus contributing to their resistance of pathogenic bacteria and enhancing their immunity (Gao et al., 2008). Further, studies have also reported that this immunity induced by YC is passed on to offspring and reduces symptoms of coccidiosis infection (Lu et al., 2019; Sun and Kim, 2019). In addition, some previous studies have shown that YC administration positively affects poultry laying performance and intestinal health (Zhang J.C. et al., 2020). We suspect that this is because of improvements within the intestinal microbiota community, which then increase the proportion of beneficial microorganisms and inhibit the reproduction of harmful microorganisms. However, at present, there is insufficient research on the influence of YC supplementation on the productive and reproductive performance of aged layers, and it is unclear whether YC affects these parameters by regulating the layers’ intestinal microbiome. Therefore, this study aimed to explore whether YC supplementation can improve laying performance, egg quality, and reproductive performance of breeder-aged layers by improving the intestinal microbial flora structure.

## Materials and Methods

### Birds, Diets, and Management

The present experiment was conducted according to the principles of the Animal Care and Use Committee of China Agricultural University. A total of 224 Hy-Line Brown laying hens (54 weeks old) were randomly assigned to two dietary treatments: a basal diet (control group, DC) and a basal diet supplemented with 2.0 g/kg of YC (Tianxiangyuan Biotechnology, Co., Ltd.), with seven replicates of 16 hens each (four birds per cage). The YC contained the following: crude protein, 53.71%; polypeptide, 12.53%; polypeptide/crude protein, 23.33%; amino acid, 1.06%; phosphorus, 7.1 mg/g; organic acid, 8.79%; pH, 4.43; and water, 46.12%. The study was conducted for an experimental period of 9 weeks (commenced from 55 weeks old), including 1 week for adaptation (54 weeks old) and 8 weeks for the experiments. The study was performed in HuaYu Poultry Breeding Co., Ltd (Handan, Hebei). All bird management was consistent with the recommendations of the Hy-Line Brown laying hen management guide. The basal diets (Table 1) comprised maize and soybean, and conformed to the Nutrients Requirements of Laying Hens of China (NY/T33-2004).

**TABLE 1:** Ingredients and nutrient composition of basal diet.

Ingredients	Percent	Nutrient level^*c*^	Percent
Corn (CP 8.3%)	64.0	ME (MJ/kg)	16.01
Soybean meal (CP 44.0%)	19.8	CP (%)	16.04
Soybean oil	0.7	CF (%)	3.24
Wheat bran	3.0	Methionine (%)	0.24
Limestone	9.5	Lysine (%)	0.70
Calcium hydrogen phosphate	1.00	Calcium (%)	3.49
Sodium chloride	0.30	Total P (%)	0.32
DL-Methionine (98%)	0.10		
L-Lysine HCL (78%)	0.07		
Vitamin premix^*a*^	0.03		
Mineral premix^*b*^	0.20		
Choline chloride (50%)	0.15		
Phytase	0.02		
NSP enzyme	0.02		
Total	100.0		

*^*a*^Supplied per kilogram of diet: vitamin A, 13,500 IU; vitamin D3, 4,500 IU; vitamin E, 75 IU; vitamin K3, 3.6 mg; vitamin B1, 3.0 mg; vitamin B2, 9.24 mg; vitamin B6, 6.0 mg; nicotinic acid, 66 mg; pantothenic acid, 16.8 mg; biotin, 0.54 mg; folic acid, 2.10 mg; vitamin B12, 0.03 mg; vitamin C, 135 mg; choline, 675 mg; ethoxyquinoline, 15 mg.*

*^*b*^Mineral premix provided per kilogram of complete diet: iron, 80 mg; copper, 10 mg; manganese, 100 mg; zinc, 100 mg; iodine, 0.35 mg; selenium, 0.30 mg.*

*^*c*^ME, CP, and CF were measured values, and the other nutrients were calculated values.*

### Performance Parameters

Egg numbers and weights were collected daily. Hens were weighed individually at the beginning and end of the experiment. The average egg yield, egg weight, broken egg ratio, abnormal egg (including double yolk egg, sand-shell egg, soft-shell egg, and those with obvious malformed-shell eggs) ratio during the intervals of 55–59 and 59–63 weeks were measured. Feed consumption was recorded, and the feed conversion ratio (FCR, feed/egg, g/g) was calculated every 28 days (FCR = feed intake per replicate/total weight of eggs laid per replicate). Mortality was documented every day as it appeared.

### Egg Quality Parameters

At the end of the study, 70 eggs were randomly collected per group (10 eggs per replicate) to determine egg quality indices. Egg index and eggshell color were measured using an egg-shaped index tester and an eggshell color tester (Konicaminolta CM-2600d), respectively. The color indices L^∗^, a^∗^, and b^∗^ represent lightness, redness, and yellowness, respectively. Eggshell breaking strength was measured using a quasi-static compression device (Robotmation, Japan). Eggshell thickness was measured at three locations, the lower end, middle end, and upper end, by using a micrometer screw gauge. Albumen height, Haugh units, and yolk color were measured using an automatic egg quality analysis instrument (Robotmation EMT-5200, Japan).

### Blood Biochemical Parameters

Blood samples from the wing vein were collected from one hen per replicate at the end of the experiment. Serum was collected and stored at −20°C until analysis. The kits for analyzing Immunoglobulin G (IgG), Immunoglobulin A (IgA), Lysozyme (LZM), Malondialdehyde (MDA), Glutathione (GSH), Glutathione Peroxidase (GSH-PX), total antioxidation capacity (T-AOC), alanine aminotransferase (ALT), and aspartate aminotransferase (AST) were purchased from Nanjing Jiancheng Bioengineering Institute (Nanjing, China). The standard hemagglutination antigens H5N6, H5N8, and Newcastle disease (ND) (Qingdao Yebio Bioengineering Co., Ltd., China) were used to detect serum antibody titers using the hemagglutination-inhibition assay.

### Ileal Nutrient Digestibility

From days 52 to 56 of the experiment, 0.5% titanium dioxide, an indigestible marker, was mixed into the feed of each group. At the end of the study, eight hens were slaughtered by cervical dislocation, and their ileum samples were collected in a circular aluminum box. These were immediately held on dry ice and quickly transferred to a −80°C refrigerator for storage until analysis. In the same group, ileum contents of every two hens were mixed, dried by baking at 105°C for 24 h, removed, and then placed in a desiccator for 4 h until the weight was constant. The contents were then ground (0.5 mm screen) for later use. The samples were used for analyzing crude protein, crude fat, and gross energy according to the standard procedures of the Association of Official Analytical Chemists (2006).

### Reproductive Performance

All the hens were inseminated for two consecutive days, on the 51st and 52nd day, of the formal experiment. The semen was mixed and came from the same group of cocks to ensure that the semen quality would not affect fertilization and incubation. Breeding eggs were then collected on the 55th day. The total number of eggs produced on that day and the number of qualified breeding eggs were recorded. The breeding eggs were uniformly transferred to a commercial hatchery for incubation. On the 18th day of incubation, the eggs were illuminated, and the number of fertilized eggs in each group was recorded. The same repeating group of fertilized eggs were then placed in a string bag with a recording card. On the 21st day of incubation, the total number of nestlings and healthy nestlings were recorded. Finally, the rates of hatched eggs and fertilized eggs, the hatching rate, and the healthy bird rate were calculated.

### Intestinal Microbiome

Six birds were randomly selected per treatment at the end of the experiment. The ileal microbial genomic DNA was extracted using the QIAamp 96 PowerFecal QIAcube HT kit (5) (Cat. No. 51531) according to the manufacturer’s protocols. Quality and quantity of DNA were verified using NanoDrop and agarose gel electrophoresis. The extracted DNA was diluted to a standard concentration of 1 ng/μL and stored at −20°C until further processing. The universal bacterial V3–V4 region of the 16S rRNA genes was amplified using polymerase chain reaction (PCR) bar-coded primers 343 F (5′-TACGGRAGGCAGCAG-3′) and 798 R (5′-AGGGTATCTAATCCT-3′). PCR was performed at 95°C for 2 min, followed by 30 cycles of 95°C for 30 s, annealing at 55°C for 30 s and at 72°C for 30 s, and a final extension at 72°C for 5 min. PCR products were detected using 1% agarose gel electrophoresis. They were further purified using the AxyPrep DNA Gel Extraction Kit (Axygen Biosciences, Union City, CA, United States) and quantified using QuantiFluor^TM^ ONE dsDNA System (Promega, United States) according to the manufacturers’ protocols. The purified amplicons were pooled in equimolar concentrations and loaded on an Illumina MiSeq platform (Oebiotech, Shanghai, China). Sequencing was performed using a paired-end (2 × 300) configuration. All operations followed standard protocols. The raw data were uploaded to the National Center for Biotechnology Information’s Sequence Read Archive database (SRA accession: PRJNA675783).

### Bioinformatics Analyses

Bacterial data were subjected to bioinformatics analyses. Raw sequencing data, which were in the FASTQ format, were demultiplexed and quality-filtered using QIIME software (version 1.8.0) (Caporaso et al., 2010). First, trimmomatic software (Bolger et al., 2014) was used to pre-process the paired-end sequences and detect and remove ambiguous bases. Second, FLASH software (Reyon et al., 2012) was used to assemble paired-end reads. Reads with Q20 values greater than 75% were retained, and chimaeras in reads were removed. Removed primer sequences were subjected to the Vsearch software and clustered with clean reads (Edgar et al., 2011). A sequence similarity of 97% was used to classify and generate operational taxonomic units (OTUs). All representative sequences were annotated and blasted against the Silva database (version 123) with the RDP classifier (confidence threshold of 70%) (Wang et al., 2007). Alpha and beta diversity were calculated using QIIME 1.8 scripts. The Venn diagram and species accumulation curves were implemented using the R Vennerable and vegan packages, respectively.

Linear discriminant analysis of effect size (LEfSe) and indicator analysis were used to identify iconic representative species. LEfSe analysis^1^ was performed to identify taxonomic compositions that were significantly altered by YC treatment. A linear discriminant analysis value higher than 4.0 and alpha value for the factorial Kruskal–Wallis test with *P* value below 0.05 were selected for plotting and analysis. Then, we performed indicator species analysis, and the R indval package was used to detect potential signature OTUs.

Phylogenetic Investigation of Communities by Reconstruction of Unobserved States (PICRUSt 1.0.0) was used to predict metagenome functions of each sample based on its 16S rRNA marker gene sequences (Langille et al., 2013). We selected a closed reference OTU and used the sampled reads against the Greengenes 16S rRNA Gene Database (13.5). PICRUSt software was used to normalize the resulting OTU table and make metagenomic inferences with the Kyoto Encyclopaedia of Genes and Genomes (KEGG) Orthologs databases. STAMP software (Parks et al., 2014) was used to visualize significant differences in KEGG functional pathways at level 2 or 3 by Welch’s *t*-test using the Benjamini–Hochberg false discovery rate method. All PICRUSt analyses were performed online: https://huttenhower.sph.harvard.edu/galaxy/root.

Microbial network analysis was used to explore the relationships among bacteria. The R psych package was used to calculate the correlation coefficient and *P* value. Values of *P* > 0.05 and *r* < 0.6 were treated with 0. Then, the Gephi 0.9.2 software was used to visualize the network correlation diagram for microbes.

### Statistics Analysis

All the apparent data were analyzed using the statistical software SPSS 24.0 (SPSS Inc., Chicago, IL, United States). Normalized data were analyzed with a normal distribution test and homogeneity test of variance. Student’s *t*-test was used for the indices that passed the test; otherwise, the Wilcoxon non-parametric test was used. The measurement of the relative abundance (%) of bacteria within the microbiome at phylum and genus levels was performed using the non-parametric Kruskal–Wallis test to validate the significant difference. The results were expressed as the mean and standard error. Differences at *P* < 0.05 were considered significant, whereas *P* values between 0.05 and 0.1 were interpreted as trends.

## Results

### Laying Performance and Egg Quality

The laying performance analysis data of hens are shown in Table 2. No significant difference was observed in egg yield, egg weight, FCR, and body weight among the groups (*P* > 0.05). However, the damaged egg ratio at 55–59 weeks of age showed a significant reduction trend (*P* = 0.059). The egg quality index analysis data are shown in Table 3. During the 8-week experimentation period, the addition of YC significantly improved egg quality, thus increasing shell strength, yolk color, egg albumen, height, and the Haugh unit.

**TABLE 2:** Effect of supplemental yeast culture (YC) on performance of laying hens.

Items	Egg production, %	Egg weight, g	Damaged egg, %	Abnormal egg, %	FCR, g feed/g egg	Feed intake, g/day/hen	Body weight, kg
**Values at 55–59 weeks of age**							Initial
DC	74.1	62.2	4.9	2.8	2.3	107.2	2.1
YC	78.3	62.2	3.5	3.4	2.3	109.4	2.1
SEM	1.58	0.32	0.39	0.46	0.44	0.99	0.02
*P*-value	0.2	1	0.06	0.55	0.36	0.29	0.74
**Values at 59–63 weeks of age**							–
DC	76.5	62.6	4.4	5.3	2.4	115.3	–
YC	74.8	62.4	4.8	5.8	2.5	114.5	–
SEM	2	0.23	0.6	0.74	0.06	1.22	–
*P*-value	0.68	0.76	0.73	0.74	0.7	0.75	–
**Values at 55–63 weeks of age**							Final
DC	75.3	62.4	4.6	4.1	2.4	111.2	2.2
YC	76.6	62.3	4.1	4.5	2.4	111.8	2.1
SEM	1.56	0.26	0.42	0.57	0.04	0.98	0.03
*P*-value	0.69	0.87	0.56	0.68	0.77	0.75	0.74

*FCR, feed conversion ratio.*

**TABLE 3:** Effect of supplemental YC on the egg quality of laying hens.

Item	DC	YC	SEM	*P*-value
Egg index	1.3	1.3	0.00	0.80
L	59.2	59.5	0.38	0.67
A	18.7	18.5	0.17	0.46
B	30.1^*a*^	29.5^*b*^	0.14	0.02
Shell strength, kg/cm^2^	4.0^*b*^	4.3^*a*^	0.07	0.02
Egg weight, g	61.1	61.6	0.38	0.57
Yolk color	7.7^*b*^	8.1^*a*^	0.09	0.01
Egg albumen height, mm	5.8^*b*^	6.1^*a*^	0.07	0.02
Haugh unit	74.6^*b*^	77.0^*a*^	0.53	0.02
Eggshell thickness, mm	0.4	0.4	0.00	0.26

*^*a,b*^Different superscripts within a row means significantly different (*P* < 0.05).*

### Blood Biochemical Parameters

The blood biochemical parameters data are shown in Table 4. The serum parameters of lysozyme and T-AOC significantly increased, while AST levels significantly decreased in the YC group. However, serum antibody responses were not significantly influenced by YC addition.

**TABLE 4:** Effect of supplemental YC on the serum parameters of laying hens.

Item^1^	DC	YC	SEM	*P*-value
**Blood biochemical parameters**				
IgG, g/L	4.4	4.5	0.15	0.93
IgA, g/L	2.2	2.2	0.04	0.65
LZM, μg/mL	4.4^*b*^	5.0^*a*^	0.14	0.03
MDA, nmol/mL	5.6	5.5	0.13	0.92
GSH, mg/L	3.2	3.1	0.04	0.46
GSH-PX, active unit	619.7	594.9	10.23	0.26
T-AOC, U/mL	6.2^*b*^	6.8^*a*^	0.15	0.05
ALT, U/L	0.9	0.7	0.15	0.66
AST, U/L	196.3	176.4	5.93	0.09
**Antibody titers**				
H5-6, log_2_	7.5	6.7	0.26	0.11
H5-8, log_2_	8.8	8.5	0.19	0.40
ND, log_2_	11.5	12.0	0.22	0.27
H9, log_2_	11.5	11.8	0.19	0.40

*^*a,b*^Different superscripts within a row means significantly different (*P* < 0.05).*

*^1^IgG, Immunoglobulin G; IgA, Immunoglobulin A; LZM, Lysozyme; MDA, Malondialdehyde; GSH, Glutathione; GSH-PX, Glutathione Peroxidase; T-AOC, Total Antioxidant Capacity; ALT, Alanine Aminotransferase; AST, Aspartate Aminotransferase; ND, Newcastle disease.*

### Reproductive Performance

The reproductive performance analysis data are shown in Table 5. Results showed that YC supplementation significantly increased the hatching rate, showing an evident trend, thus augmenting the rate of fertilization (*P* = 0.075) and number of healthy chicks (*P* = 0.064). However, YC did not affect the rate of qualified eggs.

**TABLE 5:** Effect of supplemental YC on hatching rate of laying hens.

Item	Qualified egg rate, %	Fertilization rate,%	Hatching rate, %	Healthy chick rate, %
DC	88.6	87.9	87.0^*b*^	86.8
YC	88.8	93.2	94.9^*a*^	98.8
SEM	1.71	1.49	1.96	3.29
*P*-value	0.97	0.08	0.04	0.06

*^*a,b*^Different superscripts within a column means significantly different (*P* < 0.05).*

### Ileum Nutrient Digestibility

The ileum nutrient digestibility analysis data are shown in Table 6. Although YC supplementation significantly improved the digestibility of ileum crude fat, the effect on digestibility of crude protein and energy was of little significance.

**TABLE 6:** Effect of supplemental YC on the nutrient digestibility of laying hens.

Item	Crude protein, %	Energy, %	Crude fat, %
DC	77.3	76.7	64.0^*b*^
YC	79.8	77.3	81.3^*a*^
SEM	1.28	1.36	4.62
*P*-value	0.40	0.84	0.05

*^*a,b*^Different superscripts within a column means significantly different (*P* < 0.05).*

### Intestinal Bacterial Richness, Diversity, and Similarity

**FIGURE 1:**
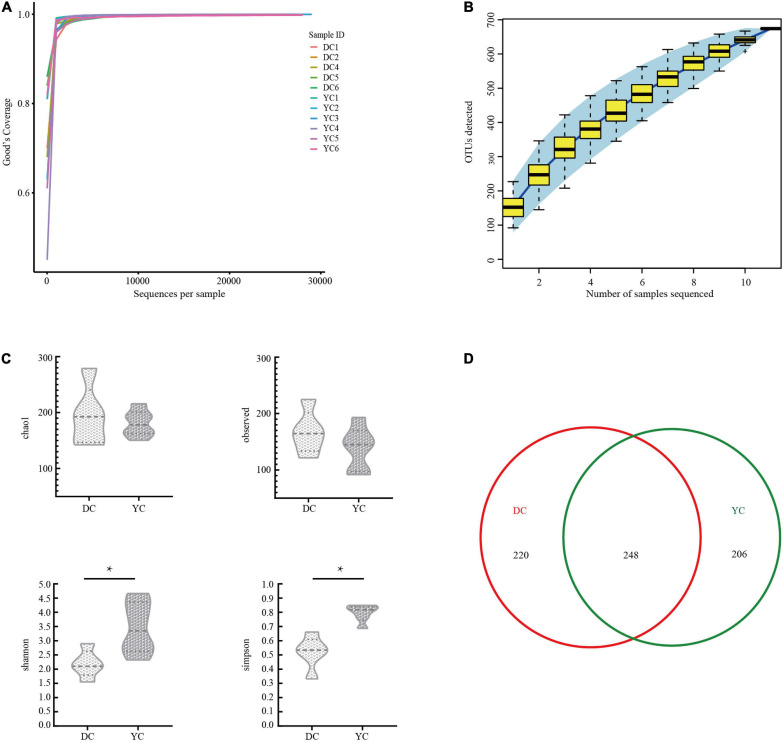
The overall description of gut microorganism in basal diet control group (DC) and yeast culture (YC) group. **(A)** The alpha diversity rarefaction curve of 16S rRNA gene sequence to estimate the rationality of sequencing depth (at 97% similarity). *X*-axis was the sequencing sampling depth, and the *Y*-axis was the corresponding Good’s Coverage index. Different sample curves were represented by different colors. **(B)** Species accumulation curve is used to estimate the rationality of sequencing sample quantity. The *X*-axis is the number of sequencing samples, and the *Y*-axis is the number of operational taxonomic unit (OTU) detected. **(C)** Alpha-diversity evaluation of ileum flora richness and evenness. **(D)** Venn diagram is used to represent the amount of OUTs that is unique or common to each group.

A total of 321,038 clean tags and 300,949 valid tags were obtained through sequencing. Through these, an average of 27,359 high-quality sequences were harvested for each ileal sample. Both the Good’s coverage rarefaction curve and the species accumulation curve indicated that the sequencing depth and sample quantity were sufficient to fully reflect the ileum microbial community composition (Figures 1A,B).

The DC and the YC groups harvested 468 and 454 OTUs, respectively. Out of these, 248 were present in both groups (Figure 1D). Alpha diversity was evaluated using the Chao1 diversity index, through observed species richness, the Simpson’s diversity index, and the Shannon diversity index (Figure 1C). Results indicated that the Shannon (*P* = 0.023) and Simpson’s diversity indices (*P* = 0.001) of the YC group were significantly higher than those for the DC group, indicating that the former group’s intestinal flora is more evenly distributed.

**FIGURE 2:**
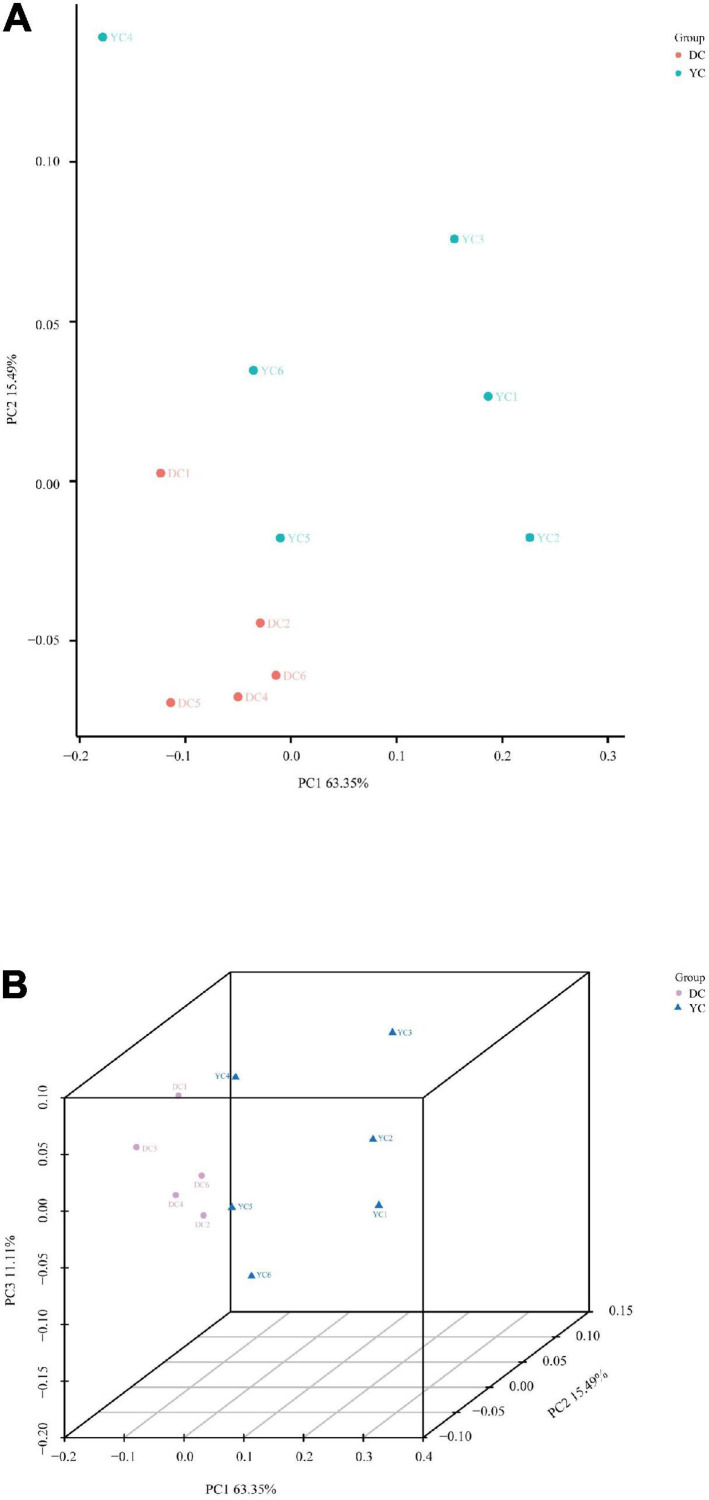
Principal coordinate analysis of ileal microbial community. **(A)** (PCoA)-2D. **(B)** (PCoA)-3D.

Beta diversity, which was based on weighted UniFrac distances, was calculated using principal coordinate analysis through both 2D and 3D plots (Figure 2). The DC and YC groups were well separated, with 63.35, 15.49, and 11.11% variation explained by principal components PC1, PC2, and PC3, respectively (ANOSIM = 0.041). Results showed that microorganism composition in the ileum of hens was significantly altered by YC supplementation.

### Ileal Microbial Community Structure

**FIGURE 3:**
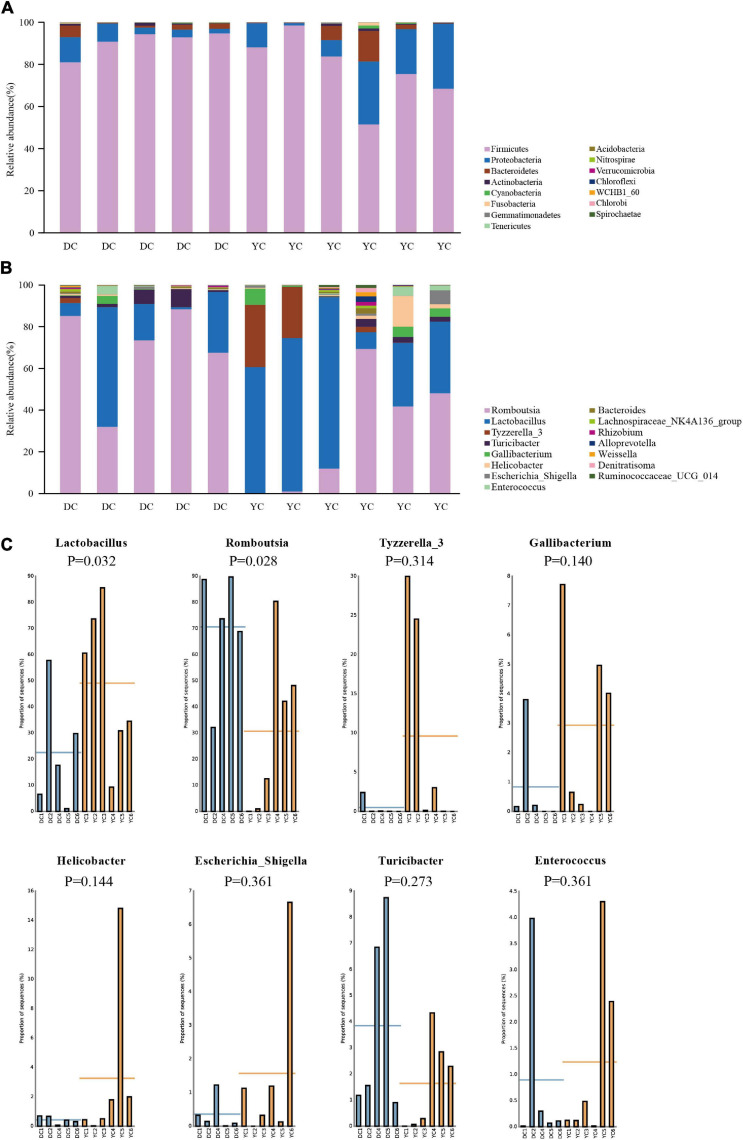
The microbial community structure in DC and YC groups. **(A)** Stacked bar chart of ileum microbial structure at phylum level. Top15 bacterial are shown in the graph. **(B)** Stacked bar chart of ileum microbial structure at genus level. Top15 bacterial are shown in the graph. **(C)** Relative abundance of microbial which was greater than 1% between DC and YC groups.

The ileal microbial community structure is shown in Figures 3A,B. Figure 3A indicates the relative abundance of microbial composition at the phylum level. Accounting for more than 98% of the bacterial community, Firmicutes, Proteobacteria, and Bacteroidetes were the predominant phyla in the DC and YC groups. However, in the DC group only, the relative abundance of Firmicutes decreased from 90.79 to 77.54% (*P* = 0.144), while that of Proteobacteria increased from 5.94% to 17.19% (*P* = 0.201). The relative abundance of other bacterial phyla did not change significantly.

Figures 3B,C and Table 7 show the relative abundance of the ileal microbial composition at the genus level. Results revealed that *Lactobacilli*, *Romboutsia*, *Tyzzerella_3*, and *Turicibacter* were the predominant genera, followed by *Enterococcus*, *Gallibacterium*, *Helicobacter*, and *Escherichia_Shigella*. Among them, the relative abundance of *Lactobacilli* increased from 14.38 to 49.83% (*P* = 0.032), while that of *Romboutsia* decreased from 58.68 to 19.54% (*P* = 0.028) when YC was added.

**TABLE 7:** Effect of supplemental YC on the Ileum bacteria of laying hens at genus level (%).

Classification levels of bacteria		Diet^1^		SEM^2^	*P*-value
	
Phylum	Genus	DC	YC		
Firmicutes	*Lactobacillus*	14.4^*b*^	49.8^*a*^	4.79	0.03
Firmicutes	*Romboutsia*	58.7^*a*^	19.5^*b*^	8.88	0.03
Firmicutes	*Tyzzerella_3*	0.4	9.1	3.23	0.31
Firmicutes	*Turicibacter*	3.3	1.1	0.80	0.27
Firmicutes	*Enterococcus*	0.4	1.0	0.34	0.36
Proteobacteria	*Gallibacterium*	0.4	2.6	0.73	0.14
Proteobacteria	*Helicobacter*	0.3	2.6	1.09	0.14
Proteobacteria	*Escherichia_Shigella*	0.3	1.2	0.45	0.36
Bacteroidetes	*Uncultured_bacterium*	0.7	1.4	0.51	0.86
–	Other	16.1	11.1	5.48	0.86

*^1^The relative abundance of Genus less than 1% are not listed. Values are means, *n* = 6.*

*^2^SEM, standard error of the mean.*

*^*a,b*^Different superscripts within a row means significantly different (*P* < 0.05).*

**FIGURE 4:**
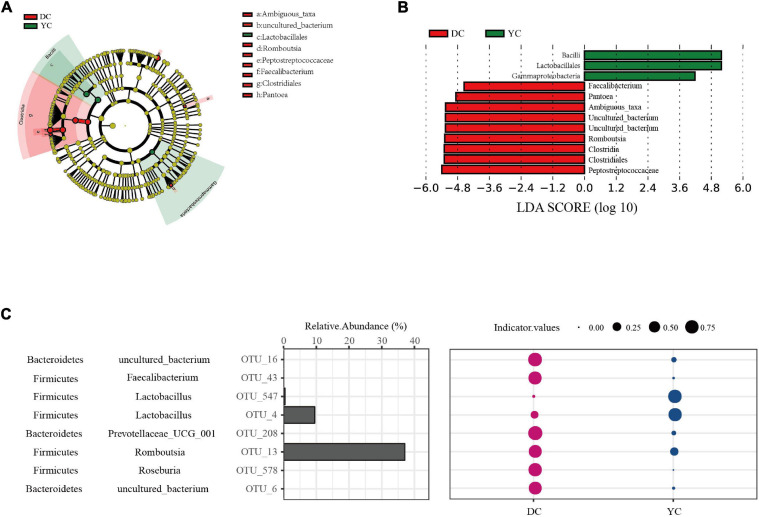
Ileal marker microbial in DC and YC groups. **(A,B).** Linear discriminant analysis coupled with effect size (LEfSe) is used to explore differences between treatment groups. **(A)** Cladogram plot of LEfSe analysis. **(B)** Histogram of LDA value distribution between DC and YC groups. **(C)** Indicator analysis is used to search for Indicator OTUs. The first column is phylum level annotation information, the second column is genus level annotation information, the third column represents the corresponding OTU, the fourth column represents the relative abundance of each OTU, and the bubble diagram represents the indicator values. Only significant (*P* < 0.05) OTUs are shown.

LEfSe analysis was conducted to determine differential bacterial form. In the YC group, Bacilli, *Lactobacilli*, and Gammaproteobacteria were significantly enriched; in the DC group, Peptostreptococcaceae, Clostridia, and *Romboutsia* were significantly enriched (Figures 4A,B).

To extend and confirm the LEfSe results, indicator analysis was performed at the OTU level (Figure 4C). Results showed that *Lactobacilli* (OTU_47, OTU_4) were the indicator species in the YC group while *Romboutsia* (OTU_13), *Roseburia* (OTT_578), *Faecalibacterium* (OTT_43), and *Prevotellaceae_UCG_001* (OTU_208) were the indicator species in the DC group.

### Ileal Microbial Network

**FIGURE 5:**
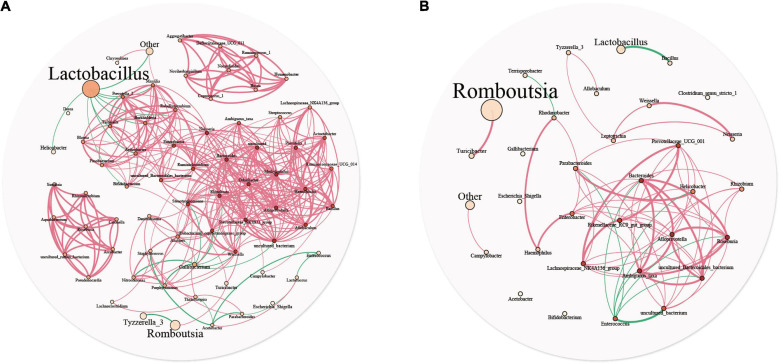
Microbial community network analysis is used to explore the relationship between the two groups. Each genus is represented by different nodes, the size of which represents the relative abundance of the genus, and the color of the nodes represents the degree. The thickness of the edges represents the correlation coefficient, the thicker, the greater of correlation. The red line represents positive correlation, and the cyan line represents negative correlation.

A microbial interaction network was used to analyze the reciprocity relationships among bacterial communities. Figure 5 describe the interaction networks of the YC and DC groups, respectively. In the DC group, the bacterial network comprised 33 nodes and 72 edges, with an average node connectivity degree of 4.364. In the YC group, the bacterial network comprised 71 nodes and 376 edges, with an average node connectivity degree of 10.592. The network complexity of the YC group was higher than that of the DC group, indicating that the microbiota of the YC group was more closely related to each other. In the YC group, *Neisseria* and *Odoribacter* showed the highest node connectivity degree (degree = 22), followed by *Alloprevotella*, *Rhizobium*, *Methylophilus*, *Prevotellaceae_NK3B31_group*, and *Bacteroides* (degree = 21). However, predominant bacteria such as *Lactobacilli* (degree = 9) and *Romboutsia* (degree = 2) had very low node connectivity degree. In the DC group, *Alloprevotella*, *Roseburia*, and *Bacteroides* had the highest node connectivity degree (degree = 11), followed by *Prevotellaceae_UCG_001*, *Rikenellaceae_RC9_gut_group*, and *Lachnospiraceae_NK4A136_group* (degree = 9). Similar to the YC group, predominant bacteria such as *Romboutsia* (degree = 1) and *Lactobacilli* (degree = 1) showed relatively low node connectivity degree in the DC group.

In the YC and DC groups, *Lactobacilli* and *Romboutsia* showed the highest proportions, respectively. The positive correlation was more than the negative correlation. Moreover, in the network interaction structure, the bacterial groups with the highest and lowest relative abundance in the intestinal tract had a lower degree of correlation with other bacteria.

### Predicted Functions of Ileal Bacterial Communities

**FIGURE 6:**
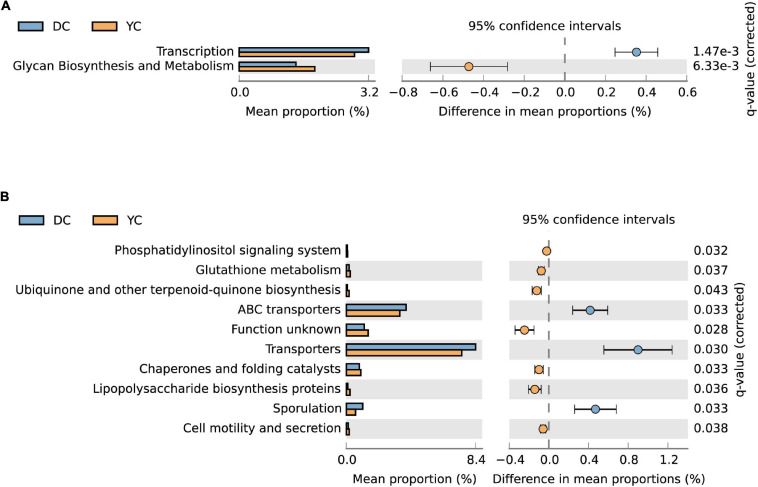
The microorganism function prediction in DC and YC groups. The second level **(A)** and third level **(B)** of Kyoto Encyclopaedia of Genes and Genomes (KEGG) pathway are shown in the extended error bar. The corrected *p*-value is listed at the right. Blue and yellow represent DC and YC, respectively.

PICRUSt analysis was used to predict metagenome functions associated with bacterial communities based on 16S rRNA sequencing data. Results showed significant differences between groups at KEGG levels 2 and 3 (Figure 6). At KEGG level 2, the glycan biosynthesis and metabolism pathways were significantly enriched by YC supplementation, while the transcription pathway was significantly downregulated. At KEGG level 3, seven pathways were enriched through YC supplementation, including the phosphatidylinositol signaling system, glutathione metabolism, ubiquinone and other terpenoid-quinone biosynthesis, chaperones, and folding catalysts, lipopolysaccharide biosynthesis proteins, and cell motility and secretion. Pathways such as ABC transporters, transporters, and sporulation were overrepresented in the DC group.

## Discussion

Yeast culture did not significantly affect the laying performance of layers, a result that was not in agreement with some previous studies. Zhang J.C. et al. (2020) reported that adding 3.0 g/kg YC to the feed of 67-week-old hens can improve their egg-laying rate and the total egg weight, and reduce the feed/egg ratio. Lu et al. (2019) found that the addition of YC alleviated laying performance loss and intestinal damage caused by *Eimeria* in broiler breeders. The variable results of the present study could be attributed to the good health of the hens used in this experiment. Additionally, some reports have indicated that differences may be due to the changes in the treatment of YC, especially factors such as administration time and period, diet, and environment (Gadde et al., 2017).

In this study, YC was found to significantly increase eggshell strength, egg albumen height and Haugh unit. However, it significantly reduced the b^∗^ value of egg shell color. In line with our findings, several previous studies have demonstrated that adding yeast or yeast extract can significantly improve egg weight, albumen height, Haugh unit, eggshell strength, and nutrient content (Zhong et al., 2016; Özsoy et al., 2018; Gaboardi et al., 2019); however, other studies have shown that dietary YC supplementation has no significant effect on eggshell strength, egg weight, albumen height, Haugh unit, and yolk color (Yalçin et al., 2008; Zhang J.C. et al., 2020). The positive effect of YC on egg quality may be associated with the presence of bioactive substances such as enzymes, vitamins, amino acids, polypeptides, and oligosaccharides in it (Jensen et al., 2008). Moreover, the presence of many carotenoids in YC, which promote pigment deposition in the egg yolk, may also be a factor in its effectiveness (Kot et al., 2019). As our results indicate improvements in egg quality, the increased hatching rate, the improved fertilization rate, and the healthy chick rate can also be associated with the egg quality and the increased eggshell strength and density, which prevent pathogenic microorganisms from entering the eggs. Moreover, YC also contains a large amount of trace elements, such as selenium and zinc that improve embryonic and early postnatal development of the aged layer (Hostetler et al., 2003; Sun et al., 2012). A previous study has shown that adding yeast bioactives significantly improves the level of IgA in the egg yolk (Lu et al., 2019), resulting in enhanced chick immunity and improved reproductive performance of the aged layers.

Hens in the late laying stage are considered to be affected by oxidative stress and ovarian aging; furthermore, an increase in lipid and protein oxidation substantially influences the normal physiology of layers (Liu et al., 2018). To counter such oxidation, T-AOC is an important integrative index that reflects antioxidant capacity (Zhang S. et al., 2020). Our results indicated that YC supplementation significantly increased lysozyme and T-AOC in serum and reduced serum AST. Similarly, a previous study showed that supplemented yeast hydrolysates tended to increase serum T-AOC (Fu et al., 2019). Thus, improvements in serum T-AOC and lysozyme activity indicated that YC administration can improve antioxidant capacity and enhance innate immunity (Min et al., 2015) of layers. AST normally exists in liver cells, and plasma AST and alanine aminotransferase contents are the most sensitive indicators of liver injury in poultry (Lumeij, 1997; Yousefi et al., 2005). Under normal circumstances, the upper limit of AST content in poultry plasma is 230 IU/L (Jones, 1999). In our study, AST content in both groups was within the normal range; however, it showed a trend of significant decrease after YC addition, which is a positive signal for liver function.

Feed digestibility and absorption gradually decreased as the age of layer hens increased (Duan et al., 2015). Thus, improving feed digestibility and availability through nutritional strategies is important to maintain constant egg production, egg quality, and health in aged hens. The results of this study showed that YC supplementation significantly improved the digestibility of crude fat, but its effect on the digestibility of energy or crude protein was of little significance. The digestion, absorption, and utilization of fat largely depend on trypsin digestive enzymes and emulsifiers (Aloulou et al., 2015). Additionally, studies have shown that the addition of YC can significantly increase the activity of duodenal enzymes in aged layers. Moreover, YC is known to contain enzymes and organic acids. Thus, our results suggest that increased fat digestion and absorption is possibly related to stimulating endogenous enzyme secretion or enzymes contained in YC (Dawood et al., 2020; Zhang J.C. et al., 2020). Intestinal microorganisms can produce bioactive substances and substantially influence health, nutrition, and immunity (Hooper et al., 2002; Ley et al., 2008; Xu M. et al., 2020). Our study showed that adding YC could alter the ileum microbial community structure in aged layers. The results of this study showed that Firmicutes, *Bacteroides*, and Proteobacteria were the dominant microbial species in the ileum, which was confirmed by other studies (Wang et al., 2018; Spring et al., 2020). After YC supplementation, the relative abundance of Firmicutes decreased (from 90.76 to 77.64%) while that of Proteobacteria (from 5.92 to 17.08%) and Bacteroidetes (from 2.31 to 3.94%) increased. The proportion of Firmicutes/*Bacteroides* (F/B) also increased (from 100.12 to 332.29). Studies have shown that the F/B ratio is related to inflammatory bowel disease (Frank et al., 2007), obesity (Turnbaugh et al., 2008), and type 2 diabetes mellitus (Remely et al., 2014). In this study, the decrease in Firmicutes and the increased F/B values indicated that YC might have a regulating effect on lipid metabolism and gut health and also reduce fat deposition in aged layers.

All the results of this study indicated that *Lactobacilli* were the dominant genus in the ileum, accompanied by a significant decrease in *Romboutsia* in the YC group. A study by Li et al. (2018) found that in day-old sika deer, *Halomonas* (48.9%), *Lactobacilli* (21.4%), and *Escherichia Shigella* (19.2%) were the dominant genera in the jejunum and ileum; with increasing age, the abundance of *Lactobacilli* began to reduce gradually from 21.4 to 6.0%. *Romboutsia* appeared and gradually became the dominant species with a relative abundance of 22.9%. Since our results suggest higher abundance of *Lactobacilli* in the intestinal tract after YC supplementation, it may be a good indicator of aged layers as *Lactobacilli* are involved in metabolic activity, such as decomposing proteins and sugars in food, synthesizing vitamins and promoting the fermentation and degradation of fat (Wu et al., 2015). Moreover, carbohydrates can be converted by *Lactobacilli* into lactic acid for further use by other bacteria (Wu et al., 2018). *Lactobacilli* have been shown to be negatively correlated with lipid metabolism. They accelerate the synthesis of lipid peroxidation metabolites and are positively related to intestinal health (Chen et al., 2020). Some experiments related to the addition of *Lactobacilli* also revealed that they have the potential to improve the performance, digestion, and feed utilization efficiency of broilers and laying hens (Saleh et al., 2017, 2020). *Romboutsia* can metabolize carbohydrates, synthesize amino acids and vitamins, and are sensitive to bile acids (Gerritsen et al., 2017). Some studies suggest that *Romboutsia* may be linked to obesity (Hu et al., 2019) and liver injury (Yu et al., 2020). Furthermore, a large number of studies have directly or indirectly proved that *Romboutsia* and *Lactobacilli* show opposing trends in relative abundance (Chen et al., 2019; Lee et al., 2019; Qiao et al., 2019; Xu S. et al., 2020). Based on these results, we can speculate that these two bacteria have especially strong competitive exclusion effects. The increased abundance of *Lactobacilli* following a decrease in *Romboutsia* may be due to polysaccharides in YC being used by *Lactobacilli* to produce a large number of short-chain fatty acids and lactic acid, which reduce intestinal pH. Another reason may be the increased shedding of *Romboutsia* due to the occupied effect of *Lactobacilli* (Baldwin et al., 2018; Qiao et al., 2018; Bunte et al., 2020).

Gut microbes can convert indigestible glycans into short-chain fatty acids such as butyrate, propionate, and acetate as nutrients for hindgut intestinal epithelial cells (Koropatkin et al., 2012; Sun et al., 2016). YC contains a large amount of glucan and mannooligosaccharides, which can induce the upregulation of glycan biosynthesis and metabolism pathways in microorganisms. Glutathione metabolism is generally considered to be related to the promotion of cell redox balance. It has antioxidant and detoxifying effects and provides a protective response (Xu et al., 2015). Glutathione-S-transferase and glutathione peroxidase are two important enzymes involved in glutathione metabolism (Schneider et al., 2016). In this study, we found that serum T-AOC content in the YC group increased significantly, which may be associated with improvement in intestinal microbial glutathione metabolic function. Ubiquinone and other terpenoid-quinone usually refer to hydrocarbon or terpenoid derivatives, and their oligomers, such as coenzyme Q10, squalene, farnesol, vitamin A, E, and K, are necessary for life activities. The upregulation of ubiquinone and other terpenoid-quinone biosynthesis pathways in YC-treated hens may be related to improvement in laying performance. Lipopolysaccharides, also known as endotoxins, are not only the main component of the outer membrane of Gram-negative bacteria but are also the main cause of inflammation and part of the natural immune response of animals. The upregulation of the lipopolysaccharide biosynthesis protein pathway after YC supplementation may be associated with an increase in lysozyme activity in the serum. Furthermore, we found that the sporulation pathway was significantly downregulated by YC. Spores are formed by bacteria in a near-dormant state. They can store the bacteria’s hereditary material in an unsuitable and harmful environment (Huang and Hull, 2017; Bressuire-Isoard et al., 2018). Metabolism in the spore state is 10 million times slower than that in normally growing bacteria. YC supplementation may contribute toward improving the original harsh ecological environment within which microorganisms live, along with increasing the vitality of bacterial communities and strengthening network relationships. ABC transporters use the energy generated through ATP binding and hydrolysis to transport various substrates on the cell membrane. They have transport and excretion roles in prokaryotes and eukaryotes, and can remove toxins and drugs from cells. Research has shown that the ABC transporter system plays a very important role in the adaptation of *Escherichia coli* to unsuitable environments (Moussatova et al., 2008; Cario, 2017). Hence, the upregulation of the ABC transporter pathway in the DC group in our study could be due to the poor ecological environment, which need to excrete a large amount of toxic substrates.

Interestingly, we found that YC supplementation substantially improved the relationships among bacteria in the ileum. An increase in *Lactobacilli* abundance in the YC group led to more complex interactions among the bacteria. Such a complex network has been suggested to increase resistance against pathogen invasions because the pathogens would have to adapt to the external environment and compete for the original ecological niche with the existing bacteria (Wei et al., 2015; Mendes et al., 2018). As the DC group had very low bacterial connectivity, and based on its function prediction, we speculated that many bacteria may be in the resting state, and the loss of one niche will substantially influence the whole microbial community. On the contrary, the YC group had very high bacterial connectivity; even when it suffered from a pathogen invasion or was missing one niche, the neighboring niches supplemented any gaps. Moreover, connectivity among bacteria limits the nutrient supply for any invasive microorganisms, causing their extinction. Therefore, a complex network of microbes shows stronger resistance to external influences.

In addition, we found that within networks of intestinal microbial relationships, bacteria that were in high abundance showed a lower degree of correlation. Furthermore, the number of positive correlation was significantly more than negative correlation in the whole network relationship. Interestingly, common probiotics such as *Lactobacilli*, *Enterococcus*, and *Acetobacter* were mostly found to be negatively correlated with other bacteria, which may be because they produce bacteriocin to inhibit the growth and reproduction of other bacteria (Corr et al., 2009). *Lactobacilli* enhance the degree of correlation between bacteria and improve the consistency of bacterial flora. An increase in *Lactobacilli* abundance after YC administration was accompanied by a more complex correlation between bacterial communities. *Lactobacilli* could thus improve the intestinal microenvironment of layers and inhibit the growth of some pathogenic bacteria. Therefore, YC supplementation can result in good health of layers.

## Conclusion

In conclusion, this study suggested that adding 2.0 g/kg YC to the diet of breeder-aged layers can improve their egg quality and reproductive efficiency. It may also be beneficial for their antioxidant capacity and systemic immunity. Moreover, it can improve the structure of aged layers’ intestinal microbial flora by increasing the abundance of *Lactobacilli* and the consistency of bacterial flora, resulting in improved feed digestion, absorption, and gut health.

## Data Availability Statement

The datasets presented in this study can be found in online repositories. The names of the repository/repositories and accession number(s) can be found below: https://www.ncbi.nlm.nih.gov/, PRJNA675783.

## Ethics Statement

The animal study was reviewed and approved by principle of the Animal care and Use Committee of China Agricultural University.

## Author Contributions

YL and XC performed the study, contributed to most of the experiments, and wrote the manuscript. WZ and DZ provided calculation and operation support. ZW, ZN, and LQ designed the study, evaluated the test details, and revised the manuscript. All authors contributed to this study and approved to submit the final manuscript.

## Conflict of Interest

DZ was employed by company Huayu Agricultural Science and Technology Co., Ltd. The remaining authors declare that the research was conducted in the absence of any commercial or financial relationships that could be construed as a potential conflict of interest.

## Footnotes

1 http://huttenhower.sph.harvard.edu/galaxy/
